# “Keep Calm and Carry On”: Structural Correlates of Expressive Suppression of Emotions

**DOI:** 10.1371/journal.pone.0016569

**Published:** 2011-01-26

**Authors:** Simone Kühn, Jürgen Gallinat, Marcel Brass

**Affiliations:** 1 Department of Experimental Psychology and Ghent Institute for Functional and Metabolic Imaging, Faculty of Psychology and Educational Sciences, Ghent University, Ghent, Belgium; 2 Clinic for Psychiatry and Psychotherapy, St Hedwig Krankenhaus, Charité University Medicine, Campus Mitte, Berlin, Germany; University of Groningen, Netherlands

## Abstract

There is a growing appreciation that individuals differ systematically in their use of particular emotion regulation strategies. Our aim was to examine the structural correlates of the habitual use of expressive suppression of emotions. Based on our previous research on the voluntary suppression of actions we expected this response-focused emotion regulation strategy to be associated with increased grey matter volume in the dorsomedial prefrontal cortex (dmPFC). On high-resolution MRI scans of 42 college-aged healthy adults we computed optimized voxel-based-morphometry (VBM) to explore the correlation between grey matter volume and inter-individual differences in the tendency to suppress the expression of emotions assessed by means of the Emotion Regulation Questionnaire (Gross & John, 2003). We found a positive correlation between the habitual use of expressive suppression as an emotion regulation strategy and grey matter volume in the dmPFC. No other brain area showed a significant positive or negative correlation with the Emotion Regulation Questionnaire scores. The association between the suppression of expression of emotions and volume in the dmPFC supports the behavioural stability and biological foundation of the concept of this particular emotion regulation strategy within an age-homogenous sample of adults.

## Introduction

Humans have the acknowledged ability of self-control. Self-control has been defined as the capacity to alter one's responses, e.g. by overriding impulses in order to bring behaviour in line with goals and standards [1], [2]. In particular the suppression of emotions serves a useful purpose in daily life. Certain situations seem to afford self-control of emotion expression, such as the need to laugh during a funeral or the experience of sexual arousal at the wrong time or place. One common form of emotion regulation is expressive suppression, which entails inhibiting the outward signs of emotion. There is a growing appreciation that individuals differ systematically in their use of particular emotion regulation strategies [3]. At the broadest level one can distinguish between antecedent-focused and response-focused emotion regulation strategies [4]. Antecedent-focused strategies refer to regulations before the response tendencies have become fully activated, whereas response-focused strategies come into play once an emotion is already underway. Gross and John [5] have designed a self-report questionnaire that assesses inter-individual differences in habitual emotion regulation, namely the antecedent-focused *cognitive reappraisal* and the response-focused *expressive suppression*. Reappraisal enfolds a cognitive-linguistic strategy that alters the trajectory of emotional responses by reformulating the meaning of a situation [6], whereas expressive suppression involves the inhibition of ongoing emotion expressive behaviour as in the so-called “poker face”. Expressive suppression has been shown to be negatively correlated with well-being [5]. Relative to the natural expression of emotions, suppression leads to increased sympathetic activation despite the concomitant decrease in somatic activity [7], [8].

Since emotion regulation strategies have been shown to be considerable stable over time [5] we set out to explore its biological underpinnings in the brain by means of voxel-based morphometry (VBM).

Since expressive suppression has been conceptualized as being strongly response-focused intervening considerably late when the peripheral physiological response of the emotion has already been triggered, we hypothesized that its neural correlates could bear resemblance to the vetoing of actions. Based on previous functional neuroimaging studies of our lab that associated the voluntary inhibition of actions [9], [10] to brain activity in the dorsomedial prefrontal cortex (dmPFC), as well as on studies on resistance to different kinds of urges [11], [12] and based on previous neuroplasticity studies that have demonstrated the impact of learning and practice on brain structure [13]–[15] we predicted that habitual use of expressive emotion suppression might be associated with an increase in volume of cortical grey matter in dmPFC.

## Methods

### Participants

42 healthy volunteers participated on the basis of written informed consent and with local ethical committee approval at University Hospital Ghent and according to the Declaration of Helsinki. No subject had a history of neurological, major medical, or psychiatric disorder. The participants (27 women and 15 men) had a mean age of 23.2 (ranging from 18 to 32) and were all right-handed as assessed by a handedness questionnaire [16].

### Questionnaire

We administered the Emotion Regulation Questionnaire (ERQ) designed by Gross & John [5] comprising the two subscales expressive suppression and cognitive reappraisal. The ERQ consists of 10 items and participants give their answers on a 7-point Likert scale with the endpoints “strongly disagree” and “strongly agree”. The expressive suppression items clearly address the tendency of participants not to show their emotions: “I keep my emotions to myself”, “When I am feeling positive emotions, I am careful not to express them.”, “I control my emotions by not expressing them.” and, “When I am feeling negative emotions, I make sure not to express them.”

### Scanning Procedure

Images were collected with a 3T Magnetom Trio MRI scanner system (Siemens Medical Systems, Erlangen, Germany) using an 8-channel radiofrequency head coil. First, high-resolution anatomical images were acquired using a T1-weighted 3D MPRAGE sequence (TR = 1550 ms, TE = 2.39 ms, TI = 900 ms, acquisition matrix = 256×256×176, sagittal FOV = 220 mm, flip angle = 9°, voxel size = 0.9×0.9×0.9 mm^3^).

### Data Analysis

Anatomical data were processed by means of the VBM5 toolbox (http://dbm.neuro.uni-jena.de/vbm) by C. Gaser and the SPM5 software package (http://www.fil.ion.ucl.ac.uk/spm). The VBM5 toolbox makes use of the segmentation algorithm of SPM5 and its implementation of a Hidden Markov Random Field approach that has been demonstrated to be superior to previous SPM versions [17]. We employed the optimized VBM protocol proposed by Good et al. [18].

We first resampled the anatomical images to a voxelsize of 1×1×1 mm^3^. Then the images were segmented into the different tissue types and the grey matter segmentations were normalized to a grey matter template. Then modulation was applied in order to preserve the volume of a particular tissue within a voxel. Modulation was achieved by multiplying voxel values in the segmented images by the Jacobian determinants derived from the spatial normalization step. In effect, the analysis of modulated data tests for regional differences in the absolute amount (volume) of grey matter. Finally, images were smoothed with a FWHM kernel of 12 mm. Then statistical analysis was carried out by means of whole brain correlation of grey matter volume with the individuals' scores on the expressive suppression and cognitive reappraisal scale of the ERQ. Sex, age and whole brain volume were entered as covariates of no interest. The resulting maps were thresholded with *p*<0.001 and cluster-size corrected by means of Monte Carlo simulation. Accordingly significant effects were reported when the volume of the cluster was greater than the Monte Carlo simulation determined minimum cluster size on whole brain grey matter volume (>149 voxels), above which the probability of type I error was below 0.05 (AlphaSim, [19]).

## Results

On the ERQ participants had a mean score of 3.04 (SD = 1.0) on the suppression factor and a mean score of 4.9 (SD = 0.99) on the reappraisal factor. Internal consistency was acceptable with Cronbachs Alpha of .71 for the suppression factor and .78 for the reappraisal factor. This is similar to the average Cronbachs Alpha that have been reported ([5]; suppression factor: .73 reappraisal factor: 0.79).

**Figure 1:**
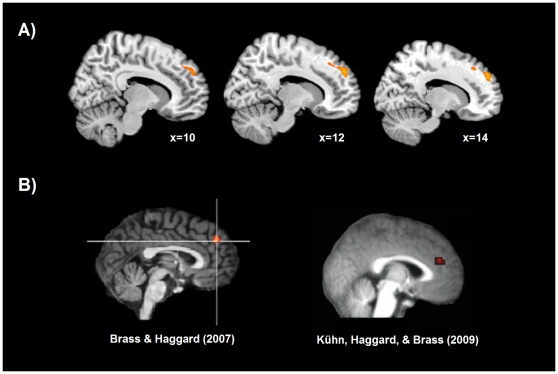
Brain regions showing a positive correlation (yellow) between expressive suppression of emotions and grey matter volume. The dmPFC (MNI coordinate 13 52 32) showed a significant correlation (p<0.001, corrected for multiple comparisons by means of Monte Carlo simulation) (A). Clusters of significant fMRI activation in previous studies during voluntary suppression of action (left: −2 41 37, right: −7 42 21) (B).

In the structural images we found a positive correlation between the inter-individual differences in expressive suppression of emotions and inter-individual differences in the grey matter volume in dmPFC (MNI coordinate: 13, 52, 32, cluster of 1364 voxels, BA 9 extending into BA 32, *Figure 1A*). No cluster of grey matter volume survived for the negative correlation with expressive suppression or for the correlations with the cognitive reappraisal score of the ERQ.

## Discussion

In line with our a priori predictions the present finding links the self-reported tendencies to use expressive suppression strategies of emotion regulation to grey matter volume in the dmPFC. More specifically, participants who habitually suppress their emotions show a significant increase in grey matter in the frontomedial wall. The fact that this self-report measure is related to structural characteristics in the brain can be seen as support of the notion that emotion regulation strategies constitute relatively stable inter-individual differences.

The location of the grey matter correlation with expressive emotion suppression is in line with our predictions based on previous functional neuroimaging research relating the dmPFC to self-control of actions and more complex urges [9]–[12]. In a study exploring vetoing of actions we asked participants to press a button at a time of their own choice but to inhibit the execution of the response on some trials that they were free to choose. When comparing voluntary inhibition with voluntary action trials, brain activation was found medially in the dmPFC (MNI coordinates: −2 41 37). In a follow-up study we used an entirely different paradigm in which the button press was rewarded in order to ensure reliable action preparation, and therefore more effortful action suppression ([10], *Figure 1B*, MNI coordinates: −7 42 21). Of note the peak coordinate of the structural correlations is found in the right hemisphere whereas the functional peak coordinates are located in the left hemisphere. One might speculate that this is in line with the traditional assumption that the right hemisphere is specialized in emotion processing [20].

Furthermore, an activation of the dmPFC has been reported during the inhibition of more complex urges such as cigarette craving [11] or loss chasing in gambling [12]. In the former study smokers were exposed to cues depicting tobacco paraphernalia and were instructed to resist the wish or to freely crave for a cigarette. The resistance to craving was associated with activity in the dmPFC. This finding suggests that dmPFC activation is not restricted to vetoing of ongoing simple actions but extends to the inhibition of more complex urges such as the urge to smoke. A study by Campbell-Meiklejohn and colleagues [12] has related functional activation of the dmPFC to loss chasing behavior which is central to pathological gambling and enfolds the continuation of gambling in order to recover previous losses. The vetoing of loss chasing was found to be related to activation in the dmPFC. Moreover the dmPFC has been shown to be engaged when an anticipated response has to be delayed until an actual Go-signal is presented [21]. This so-called proactive inhibition could be interpreted as a default state of refraining from responding, that is not necessarily a conscious process. This suggests that the association between dmPFC grey matter volume and expressive emotion suppression could reflect unconscious control processes. Taken together these functional neuroimaging findings point to the important role that dmPFC plays in self-control. Nevertheless the dmPFC is not the only brain structure that has been related to the inhibition of action. It has been suggested that the voluntary suppression of actions can be distinguished from externally instructed stopping of actions [9], [10]. One reason for that distinction is that internal and externally driven inhibition seems to rely on different neural substrates. In contrast to studies focussing on internal self-control a multitude of studies on externally triggered inhibition used stop tasks and Go/NoGo tasks in which prepotent actions had to be inhibited in response to a stimulus. These external inhibition processes have been associated with activation in the lateral prefrontal cortex, most prominently the right inferior frontal gyrus [22]–[24]. In line with this distinction functional neuroimaging studies on emotion regulation have predominantly reported the involvement of lateral but also of medial prefrontal cortex when participants were instructed to use strategies to reduce negative emotional experiences [25]–[28]. The involvement of lateral prefrontal cortex might be explained by the fact that participants were prompted to suppress emotions externally.

Based on the neural dissociation of brain regions associated with internal and external inhibition of action, the association we found between the habitual use of expressive suppression strategies and an increase of grey matter in dmPFC could suggest that emotion suppression is under internal control. Further research is needed to dissociate structural correlates of internal and external inhibition in one study. One might interpret the manifestation of expressive suppression in the dmPFC as a consequence of the internalization of societal norms, manners and mores that govern the acceptable and unacceptable times for emotional expression [29], [30]. We suspect a relationship between the functional involvement of dmPFC in the voluntary suppression of actions and the increases in grey matter volume in dmPFC related to the habitual use of expressive suppression strategies. In accordance with this assumption previous studies have demonstrated the impact of learning and practice on brain structure as e.g. in taxi drivers [13], musicians [14] and in participants who were trained in juggling [15]. Several neuroplasticity studies have shown overlap between functional plasticity and structural plasticity suggesting practice induced grey matter changes [31], [32]. However, the present finding cannot rule out that the increased dmPFC volume in subjects with expressive suppression strategies is an a priori condition rather than a consequence of behaviour.

In order to transfer our present finding of an association between expression control and grey matter volume in the dmPFC to self-control in general further research is needed to examine suppression exerted on different targets. Future studies might consider relating inter-individual differences in the suppression of the feeling and thoughts rather than the outward expressions that accompany emotions to differences in grey matter volume. Moreover future studies might elucidate the relationship between functional correlates of emotion regulation and inter-individual differences in dmPFC volume.
